# Observations on the Foot and Its Ailments1Read at the Tennessee Veterinary Medical Association, at Nashville, November 8, 1899.

**Published:** 1900-01

**Authors:** T. W. Scott

**Affiliations:** Clarksville, Tenn.


### OBSERVATIONS ON THE FOOT AND ITS AILMENTS.[1]

By T. W. Scott, V.S.,
CLARKSVILLE, TENN.

The feet of solipeds are the most important organs of locomotion ; and on locomotion depends the whole value of these animals. The idea of a good horse with poor feet is a misnomer. Then if the feet are in reality such important members, rendering the animal practically useful or worthless, according to their soundness or unsoundness, it is certain that the care and treatment of the feet are specifically in the province of the veterinarian. The feet of horses are subject to a multitude of injuries and diseases,

[1] Read at the Tennessee Veterinary Medical Association, at Nashville, November 8, 1899.

and I shall not in this short paper attempt to deal with or even to speak of them all; but I shall confine myself more particularly to the simpler diseases and injuries to which these members are heir. I shall attempt to show or point out, in the first place, some of the injuries and diseases which result from errors or mistakes in shoeing.

It would be too much to require that veterinarians should be expert horseshoers any more than that physicians should be first-class shoemakers; but horseshoeing is the orthopedics of veterinary science, and all practitioners should be familiar with the proper application and adjustment of the shoes. Shoes are adjusted to the feet of horses for the sole purpose of protection. That is the object and end of shoeing. To preserve the feet intact and in health, so that the animal can best serve its master, is the use and purpose of shoeing. Horseshoeing, therefore, resolves itself into an exceedingly important science, and requires brains and skill in its correct and proper performance.

I shall now enumerate some of the injuries and ailments which follow the improper application of shoes; and when I use the term application of shoes I mean the preparation of the foot for the shoe, the preparation of the shoe for the foot, and everything necessary for this somewhat complex operation. Under this heading I shall classify: nail-pricks, corns, burns, or blisters of the sensitive sole from a hot shoe, brittle wall, injuries to sensitive sole from pressure or uneven pressure of shoe, quarter-cracks, toe-cracks, side bones, decayed, wasted, or atrophied frog, contraction of the foot, vertical or perpendicular walls, and interfering, and diseases of the structures of the limbs by reason of irregularities and imperfections in the fitting of the shoes or the manner of preparing the feet.

Nail prick and the injury and consequent lameness produced is a frequent result of shoeing. This is an accident in which the smith should be indulged to some extent, as there are many mitigating circumstances, and the smith should not always be condemned for it, as there are walls which are so badly broken away, and what remains is so thin, that it is practically impossible to drive the nails so that they do not press upon or even penetrate the sensitive laminæ. But there are many nail pricks resulting from carelessness or incompetence on the part of the smith. Proper and expert driving is an art, and the accomplished floor-man can place his nail and tap it, and can tell in an instant by the sound and feel of it under his hammer whether it is passing through sound horn or deviating into the sensitive part. Of course, if the nail presses hard or penetrates the sensitive structures, the animal will tell him very quickly, but a nail may go close and the animal not evince pain at the time, and it may be a day or two, perhaps, before lameness results. Then the veterinarian may be asked to locate the lameness, and this is usually not difficult. And here I want to digress a little while I admonish the veterinarian to have a care, and to be exceedingly careful not to pass the foot without scrutinizing and examining it very particularly, for it is a sad blow and an awful smirch upon the reputation of the veterinarian whenever he overlooks a lesion, no matter how slight, in the foot; for in a few days the animal may be sent to the forge, and the shoer removes the shoe, and by manipulation reveals and points out the real injury or lesion.

The veterinarian should always carry a hoof searcher, and if he cannot satisfy himself fully he should go with his patient to the forge, and there have it carefully examined, or, better still, I think it well that veterinarians should operate a shoeing forge wherever it is practicable. I think it will result in good to the profession of veterinary surgery, good to the horseshoeing industry, and, lastly, an inestimable benefit to the whole equine race.

With reference to the treatment of nail pricks, little need be said. Remove the offending object, dilate the opening, wash out well with a good antiseptic solution, then use Lugol's solution of iodine injected well up into the hole. The foot may be poulticed or soaked in warm water a few times, and usually the shoe can be adjusted in a few days again.

Corns are of two varieties, namely, hard or dry corns, and suppurating corns. They are a direct result of ill-fitting shoes, and especially when the latter have high heel calkings. They are, of course, frequently caused also by the carelessness and negligence of the owner in allowing the shoe to remain on too long until the wall has grown over the shoe and forces the shoe in at the heel, and the consequent pressure, as well as from dirt and sand working under the shoe. But a corn is more frequently produced by narrow fitting, fitting too close at the heel, and again by ill-fitting shoes. For instance, the shoe may bear more heavily at this point than on the balance of the wall, and result in a bruise or corn. The term used by farriers with reference to this is that the shoe is "lying dead" at this point, and is very expressive. Corns frequently produce more or less lameness, and the veterinarian is consulted, and the remarks made above apply with equal emphasis here.

Of course, all foot lameness and injuries present certain characteristic symptoms, but they all present likewise individual symptoms, and the practitioner must locate the point or spot if possible, and in the first place the foot must be critically examined. Nothing inspires more confidence in a client's mind than when the animal under question is walked or trotted off before the examiner, and he remarks at once :

"The lameness is on the right (or the left side, as the case may be), and it has the indications of foot lameness. We will, therefore, examine that member."

The foot is picked up and cleaned out, and the searcher applied. When a corn, the animal evinces pain in either heel and the parts are dry and hot. The examiner has the shoe removed, the dry horn cut away, and the corn presents itself to view, and the veterinarian scores a point or victory, so to speak. For every time that the practitioner gives ocular demonstration of his skill he has won a victory over skepticism, for nearly every client in this region is a skeptic so far as believing that the veterinarian possesses skill or special wisdom, as I suppose you have all doubtless observed.

Corns need not necessarily lay an animal up, for by the proper application of the shoe or by applying a proper shoe correctly the animal can resume work almost at once. Suppurating corns, besides requiring a proper shoe, require also proper treatment, such as opening up well and allowing the escape of pus, washing out with peroxide of hydrogen or other antiseptic, and preventing the collection of pus thereafter. With reference to the character of the shoe for the relief of corns, I think a good bar shoe, taking the weight entirely off the affected quarter, the shoe *par excellence*, so to speak ; or the ordinary shoe with the application of the Davy hoof-pad, of which I will present a picture, not having the pad to show. These contrivances are excellent for the relief of corns, but they do not cure, and I believe result in some injury to the feet. I do not like rubber appliances to horses' feet. I despise rubber horseshoes and all rubber pads that cover the sole. They cause dryness and hardness of the foot, with their attendant evils. I take occasion here, also, to remark that I regard all leather soles or rubber soles placed under the shoe and held by the shoe as a positive injury to the horse's feet. They exclude air from the foot, they increase the heat to it, they prevent evaporation and elimination. Pardon me, also, while I pay my respects at this juncture to what I term the vicious practice of oiling the hoofs of horses.

Oiling the hoofs is an injury to them, resulting in dryness, rendering brittle, preventing breathing, evaporation, elimination of impurities and excretions, which the foot will naturally rid itself of for its own protection and benefit. The hoof oiled or smeared with grease will accumulate more dust, dirt, and filth in a given time than one can well imagine. Oiling is practised through ignorance and by smart horseshoers to cover up defects and blunders in shoeing.

I also condemn all hoof ointments which many veterinarians are in the habit of using and prescribing for mercenary purposes. I go so far as to say that the hoof should never be oiled or greased except after soaking. Then and only then is it beneficial. At that time it prevents evaporation, and retains the moisture in the foot, for which the soaking was inaugurated.

I shall not dwell longer on the subject of corns, but shall hope that my remarks may evoke extensive discussion by the members present.

Burns and blisters of the sensitive sole from the application of hot shoes are not of very frequent occurrence, but do occur now and again when the soles are very thin and the fitter does not exercise care in applying the hot shoe, but keeps it applied in the hot state too long or presses too hard. Serious damage is sometimes done in this way, and usually the animal will evince pain at the instant, but there are cases in which the effects are not apparent for a day or so. Then the veterinarian may be called, and may have some difficulty in explaining the exact character of the lesion. The animal will go very lame, and will resemble laminitis if both feet are burnt; if only one, laminitis of one foot, and the cause may be difficult to explain. In these remarks it is not intended that any aspersion or anything derogatory to hot fitting is meant; for I am an advocate of judicious hot fitting, and claim that it is the only practical manner of fitting a shoe, and does no harm when done with proper care and discretion. Cold fitting is impracticable and requires much more time. You can easily understand that it is next to impossible to fit two cold surfaces so that they are the exact counterparts of each other, or so that the two surfaces are in exact apposition. Hunting and Fleming both claim that hot fitting is the only practical fit, and when done with skill and discretion results in no harm if not benefit to the parts. The hot shoe is applied to burn down the little elevations or eminences which are bound to remain after the foot has been prepared as nearly as possible by the smith with rasp and knife, and if the shoe is red, or even at white heat and applied only for an instant, it burns down these elevations and prominent points, bringing the shoe in perfect apposition with the foot, softening the hoof underneath, rendering cutting easier, and facilitating the driving of the nails. Then a certain quantity of water is extracted from the hoof, drying it somewhat, thereby holding the nails more securely. But remember I do not say that indiscriminate hot fitting is wise. The smith must have discretion and knowledge of the parts upon which he is operating.

Brittle walls are often produced by too much rasping of the outer wall, by using nails that are too large, and especially by oiling the feet or by excessive soaking or poulticing, and can be corrected by removing the causes.

Injuries of the sensitive sole are produced by uneven pressure of the shoe, the shoe lying dead at a given point and pressing upon the sole. There is no objection to the shoe pressing upon the sole when the latter is strong and thick, but when it is thin and weak the shoe should be seated out or concaved so that it cannot press on the sole, or the wall should not be removed to the extent that the sole will come in contact with the shoe. Pressure upon thin sole gives rise to lameness, and the veterinarian must determine what the lesion is, and remove it.

Toe- and quarter-cracks are frequently a source of much lameness, but generally come under the supervision of the horseshoer or farrier. They occur in weak-quartered, weak-walled feet, and may be the result of ill-fitting shoes. They can be grown out by proper firing and blistering and adjusting a thin brass plate over the crack with screws, together with the use of a bar shoe, which will invariably prevent lameness and render the animal serviceable.

Wasted frog, perpendicular or vertical walls, and contracted feet are caused by allowing the shoes to remain on too long, by improper preparation of the foot for the shoe, etc.

In shoeing horses it is always of vital importance to prepare the foot so that the frog shall have its function. It should rest upon the ground and receive the jar or shock, and should therefore relieve the balance of the foot, giving it spring and elasticity and preventing slipping ; and any manner of shoeing that interferes with this is wrong and is generally easily corrected.

There are many other accidents and ailments to the feet of horses incident to shoeing which I should like to have touched upon, but the paper is now too voluminous, and I shall trust that you will have anticipated all these.

